# Influence of Body Mass Index and Gender on Stigmatization of Obesity

**DOI:** 10.1007/s11695-020-04895-5

**Published:** 2020-08-09

**Authors:** Christian Tapking, Laura Benner, Matthes Hackbusch, Svenja Schüler, Danny Tran, Gregor B. Ottawa, Katja Krug, Beat P. Müller-Stich, Lars Fischer, Felix Nickel

**Affiliations:** 1 Department of General, Visceral and Transplant Surgery, University of Heidelberg, Im Neuenheimer Feld 110, 69120 Heidelberg, Germany; 2 Department of Hand, Plastic and Reconstructive Surgery, Microsurgery, Burn Trauma Center, BG Trauma Center Ludwigshafen, University of Heidelberg, Ludwig-Guttmann-Strasse 13, 67071 Ludwigshafen on the Rhine, Germany; 3 Institute of Medical Biometry and Informatics, University of Heidelberg, Im Neuenheimer Feld 130.3, 69120 Heidelberg, Germany; 4 School of Medicine, University of Texas Medical Branch, Galveston, TX USA; 5 Coordination Centre of Clinical Trials, University Hospital of Heidelberg, Im Neuenheimer Feld 110, Heidelberg, Germany; 6 Department of General Practice and Health Services Research, University Hospital of Heidelberg, Vossstr. 2, 69115 Heidelberg, Germany; 7 Department of Surgery, Hospital Mittelbaden, Balger Strasse 50, 76532 Baden-Baden, Germany

**Keywords:** Obesity, Education, Stigmatization, Stereotypes, Fat phobia scale

## Abstract

**Background:**

Stigmatization and discrimination of people with obesity due to their weight are a common problem that may lead to additional weight gain. This study evaluated the influence of different parameters on the stigmatization of obesity.

**Material and Methods:**

Participants of six groups (general population, patients with obesity, medical students, physicians, nurses in training and nurses; *n* = 490) answered the short-form fat phobia scale (FPS) between August 2016 and July 2017. The influence of body mass index (BMI), gender and other factors on total scores and single adjective pairs was analyzed.

**Results:**

A total of 490 participants were evaluated. The total mean FPS rating was 3.5 ± 0.6. FPS was significantly lower (more positive) in participants with obesity (3.2 ± 0.7) compared with participants without obesity (3.5 ± 0.5, *p* < 0.001). Individuals with obesity and diabetes rated the FPS significantly lower (more positive), whereas age and gender did not have a significant influence. Participants with obesity linked obesity more often with good self-control (*p* < 0.001), being shapely (*p* = 0.002), industrious (*p* < 0.001), attractive (*p* < 0.001), active (*p* < 0.001), self-sacrificing (*p* < 0.001) and having more willpower (*p* < 0.001) than the participants without obesity. Females rated more positive in shapely versus shapeless (*p* = 0.038) and attractive versus non-attractive (*p* < 0.001) than males.

**Conclusions:**

The present study shows that stigmatization of obesity is present in medical professionals as well as the general population. People affected by obesity characterized other people with obesity more positively (e.g. attractive or active), whereas people without obesity linked negative characteristics with obesity. Gender had an influence only on single items of FPS but did not affect overall stigmatization of obesity.

## Introduction

As recently reported by the National Center of Health Statistics, the prevalence of obesity in adults in the USA increased from 30.5 to 42.4% from 1990 through 2017–2018 [1]. Individuals with obesity are generally more likely to face social problems and restrictions due to their weight compared with individuals without obesity. This includes stigmatization in healthcare, and during their education [2–6]. It has also been reported that people with obesity may experience denial of employment due to body weight or size [7]. Global stigmatization of obesity increased over the past decades, also in healthcare providers [8]. It is a growing concern given the increasing prevalence of obesity [4, 9–12]. The society often considers obesity a “lifestyle-disease” and individuals with obesity are regarded to be responsible (“their own fault”, “are inactive”, “don’t have self-control”) [13–15]. Stereotypes connected with obesity were identified in former studies. Patients with obesity were considered to be lazy, noncompliant, unsuccessful, unintelligent and lacking of self-control [2, 13]. The opinion that obesity is a question of self-responsibility and little self-control for their weight is spread in the media as well [16]. This stigmatization is widespread in the society and can affect psychological and physical health [17]. Not limited to health, obesity stigma has been shown to impact socioeconomic status resulting in inequality, particularly in women and middle-aged individuals [18]. It has been shown that perceived stigmatization of people with obesity in the USA has increased by 66% since 1995 and is now on par with racial discrimination [10]. This common view leads to a widespread “fat phobia” in society and even within young professionals [4, 19, 20].

Stigmatism towards obesity was reported to be common in (future) medical professionals [20]. Physicians assume obese people to have poor hygiene, poor compliance and be unlikely to follow instructions such as taking their medication regularly [21, 22]. Studies have shown that medical students rated patients with obesity more often as ugly, lazy or sloppy [23, 24]. A recent meta-analysis reported the pooled prevalence of perceived discrimination towards weight to be 19.2% in people with a BMI between 30 and 35 kg/m^2^ and 41.8% in people with a BMI greater than 35 kg/m^2^, respectively [25]. These stigmatizations can impair the patients and individuals with obesity psychologically and make them more vulnerable to depression, isolation or economic hardship and this can even lead to overeating and a sedentary lifestyle [26].

People with obesity who are exposed to stigma may be vulnerable to psychological impairments such as depression, which could contribute to health outcomes such as cardiovascular diseases [26]. Initially, they may internalize the stereotypes and stigmas and develop an internal weight bias or self-stigma. This self-stigma results in low self-esteem and embarrassment about their weight. These individuals then suffer from dysregulated eating and poor weight management behaviours which eventually leads to increased binge eating and a poorer self-body image [27]. These problems continue to compound and lead to increased risk of psychologic problems such as depression, binge eating and anxiety [28]. Patients with obesity with the associated psychological problems are at risk for impaired and suboptimal health that leads to increased healthcare utilization [29]. However, it remains unknown how demographics such as age, profession, being obese and having other comorbidities influence the stigmatization of obesity. Furthermore, present studies generally draw an overall picture without addressing specific areas or characteristics where stigmatization is present, whereas the present study evaluates both healthcare and non-healthcare workers and other areas of possible influence. It is especially important to evaluate how medical professionals and people that suffer from obesity themselves see other patients with obesity. Additionally, the present study deals with the impact of various demographics on attitudes towards obesity. It is important to draw a more detailed picture of how people with obesity are seen in the society in order to overcome stigmatization and negative attitudes, which may prevent these individuals to receive appropriate medical care.

The present study aimed to analyze factors influencing the stigmatization towards obesity using the fat phobia scale (FPS) with focus on the influence of the participants’ body mass index (BMI), gender and other demographical characteristics. The present study is a secondary analysis of a randomized trial evaluating the influence of video teaching on stigmatization towards obesity in comparison to other chronic diseases [20].

## Material and Methods

Between August 2016 and July 2017, individuals with different previous knowledge about obesity took the short-form FPS questionnaire and were asked about demographics such as weight, age, gender and comorbidities. The participants were individuals of the general population, patients with obesity, medical students, doctors, nurses in training and nurses. The participants were recruited in public places (general population), in an outpatient obesity clinic at the Hospital University Heidelberg (patients with obesity), during medicine seminars and lectures at the Medical Faculty of Heidelberg (medical students), in teachings in the nurses´ academy at Heidelberg and Baden-Baden (nurses in training) and at medical congresses and advanced training sessions (physicians and nurses). For further analysis, we defined people with obesity if they had a BMI ≥ 30 kg/m^2^ (according to self-reported height and weight) or they originated from the group of patients with obesity.

### Fat Phobia Scale

In 1995, Robinson et al. created the FPS after defining fat phobia as the pathological fear of fatness associated with negative characteristics and stigmas towards individuals with obesity [30]. The FPS is a 50-item questionnaire that asks individuals to associate specific qualities with a person with obesity [30]. The short version developed by Bacon et al. that was used for this study includes 14 pairs of adjectives (one negative and one positive, e.g. active vs. inactive) with a ranking system of 1 (very positive) to 5 (very negative) for each [31]. To complete the shortened FPS, the participants were asked to rank a 42-year-old woman with a BMI of 31.9 kg/m^2^ (weight: 90 kg, height: 1.68 m) against those 14 pairs of adjectives. Averaging the value for the 14 single adjectives yields the value for the FPS.

### Statistical Analysis

The characteristics of the study population are presented separately for the participants with and without obesity by mean and standard deviation or absolute and relative frequencies. Differences between the participants with and without obesity were compared by two-sided Welch tests for continuous outcomes and by chi-squared tests for categorical outcomes. Using multiple linear regression models, the influence of gender, obesity, age and diabetes mellitus on the FPS or its single items, respectively, was examined. Missing values were not imputed. This was for example the case in participants that did not specify their gender (*n* = 12, 2.4%). Due to the exploratory character of the study, no multiplicity adjustment was conducted, and *p* values are interpreted in a descriptive manner. Statistical analyses were conducted using R version 3.6.1 [32]

## Results

A total of 490 participants answered the fat phobia scale. Of these, 81 (16.5%) people from the general population, 82 (16.7%) patients visiting the obesity outpatient clinic, 76 (15.5%) medical students, 84 (17.1%) physicians, 89 (18.2%) nurses in training and 78 (15.9%) nurses were evaluated. In average, participants with obesity (30.0 ± 11.5 years) were significantly younger than participants without obesity (43.5 ± 14.1 years, *p* < 0.020) but there were no differences regarding gender. General characteristics of the study population are shown in Table 1.

**Table 1:** General characteristics of the study group

Variable baseline	Obesity			*p* value
	Total	No	Yes	
*n* (%)	490 (100)	380 (77.6)	110 (22.4)	
Age				
Mean (SD)	33.0 ± 13.4	30.0 ± 11.5	43.5 ± 14.1	< 0.001^1^
Body mass index				
Mean (SD)	26.0 ± 8.4	22.7 ± 2.8	37.7 ± 10.6	< 0.001^1^
Gender				
Male, *n* (%)	169 (34.5)	133 (35.0)	36 (32.7)	0.734^2^
Female, *n* (%)	309 (63.1)	239 (62.9)	70 (63.6)	
Not specified	12 (2.4)	8 (2.1)	4 (3.6)	
Fat phobia scale				
Mean (SD)	3.5 ± 0.6	3.5 ± 0.5	3.2 ± 0.7	< 0.001^1^
Study group, *n* (%)				< 0.001^2^
General population	81 (16.5)	78 (20.5)	3 (2.7)	
Obese patients	82 (16.7)	0	82 (74.5)	
Medical students	76 (15.5)	76 (20.0)	0	
Physicians	84 (17.1)	79 (20.8)	5 (4.5)	
Nurses in training	89 (18.2)	82 (21.6)	7 (6.4)	
Nurses	78 (15.9)	65 (17.1)	13 (11.8)	

*n*, number; *SD*, standard deviation

^1^Welch ANOVA

^2^Chi-squared test

### Total Fat Phobia Scale

The mean of the total fat phobia scale ratings was 3.5 ± 0.6 and was significantly lower (more positive) in participants with obesity (3.2 ± 0.7) compared with participants without obesity (3.5 ± 0.5, *p* < 0.001, Table 1).

In the linear regression analysis with the dependent variable “total fat phobia scale”, individuals with obesity and those suffering from diabetes mellitus rated the FPS significantly lower (more positive). The participants’ age and gender did not have a significant influence on total FPS ratings (Table 2). Other comorbidities than diabetes did not yield any relevant associations.

**Table 2:** Regression analysis of total fat phobia scale

	*B*-value	CI	Std. Error	*p* value
(Intercept)	3.678	3.509, 3.847	0.086	< 0.001
Gender female (reference male)	− 0.051	− 0.156, 0.055	0.054	0.346
Obese	− 0.357	− 0.493, − 0.222	0.069	< 0.001
Age	− 0.001	− 0.005, 0.003	0.002	0.602
Diabetes Mellitus	− 0.153	− 0.254, − 0.051	0.052	0.003

*B-value*, estimate; *CI*, confidence interval

### Single Adjective Pairs

Linear regression analyses were also performed for each adjective pair (Table 3). Participants with obesity linked other individuals with obesity more often with good self-control (*p* < 0.001), being shapely (*p* = 0.002), industrious (*p* < 0.001), attractive (*p* < 0.001), active (*p* < 0.001), strong (*p* = 0.004), self-sacrificing (*p* < 0.001), fast (*p* < 0.001), having more willpower (*p* < 0.001) and endurance (*p* < 0.001). Participants with female gender rated more positive in shapely versus shapeless (*p* = 0.038) and attractive versus non-attractive (*p* < 0.001). There were no gender differences in other adjective pairs. Having diabetes was a significant parameter for more positive rating regarding self-control (*p* = 0.012), attractiveness (*p* = 0.022), industriousness (*p* = 0.004), being fast (*p* = 0.036), active (*p* = 0.015) and strong (*p* = 0.014).

**Table 3:** Regression analysis of individual adjective pairs of the fat phobia scale

	*B*-value	CI	Std. Error	*p* value	*B*-value	CI	Std. Error	*p* value
	Good self-control vs. poor self-control				Shapely vs. shapeless			
(Intercept)	3.497	3.258, 3.735	0.121	< 0.001	3.951	3.659, 4.242	0.148	< 0.001
Gender female (reference male)	0.019	− 0.129, − 0.168	0.076	0.797	− 0.192	− 0.373, − 0.01	0.092	0.038
Obese	− 0.368	− 0.559, − 0.177	0.097	< 0.001	− 0.365	− 0.597, − 0.132	0.118	0.002
Age	− 0.004	− 0.01, 0.002	0.003	0.244	− 0.002	− 0.01, 0.005	0.004	0.524
Diabetes mellitus	− 0.182	− 0.325, − 0.04	0.072	0.012	− 0.141	− 0.314, 0.033	0.088	0.113
	Industrious vs. lazy				Attractive vs. non-attractive			
(Intercept)	3.713	3.346, 3.961	0.126	< 0.001	3.957	3.676, 4.237	0.143	< 0.001
Gender female (reference male)	− 0.077	− 0.231, 0.077	0.078	0.325	− 0.434	− 0.608, − 0.259	0.089	< 0.001
Obese	− 0.490	− 0.688, − 0.292	0.101	< 0.001	− 0.524	− 0.749, − 0.299	0.115	< 0.001
Age	− 0.009	− 0.015, − 0.003	0.003	0.005	0.001	− 0.006, 0.008	0.004	0.740
Diabetes mellitus	− 0.218	− 0.366, − 0.07	0.075	0.004	− 0.195	− 0.363, − 0.028	0.085	0.022
	Willpower vs. no willpower				Self-confident vs. not self-confident			
(Intercept)	3.696	3.434, 3.958	0.133	< 0.001	3.468	3.186, 3.751	0.144	< 0.001
Gender female (reference male)	− 0.118	− 0.281, 0.046	0.083	0.158	− 0.014	− 0.19, 0.162	0.090	0.876
Obese	− 0.384	− 0.594, − 0.175	0.107	< 0.001	− 0.076	− 0.3, 0.154	0.115	0.526
Age	− 0.009	− 0.01, 0.004	0.003	0.005	− 0.001	− 0.008, 0.007	0.004	0.867
Diabetes mellitus	− 0.070	− 0.226, 0.087	0.080	0.381	− 0.161	− 0.329, 0.08	0.086	0.061
	Fast vs. slow				Having endurance vs. having no endurance			
(Intercept)	3.861	3.616, 4.105	0.125	< 0.001	4.241	3.978, 4.505	0.134	< 0.001
Gender female (reference male)	− 0.069	− 0.222, 0.084	0.078	0.376	− 0.096	− 0.26, 0.068	0.083	0.250
Obese	− 0.473	− 0.669, − 0.278	0.100	< 0.001	− 0.377	− 0.587, − 0.166	0.107	< 0.001
Age	− 0.003	− 0.009, 0.004	0.003	0.421	− 0.011	− 0.017, − 0.004	0.003	0.001
Diabetes mellitus	− 0.157	− 0.304, − 0.011	0.075	0.036	− 0.077	− 0.234, 0.08	0.080	0.337
	Active vs. inactive				Strong vs. weak			
(Intercept)	3.738	3.483, 3.994	0.130	< 0.001	2.955	2.706, 3.204	0.127	< 0.001
Gender female (reference male)	− 0.124	− 0.283, 0.034	0.081	0.123	− 0.013	− 0.168, 0.142	0.079	0.867
Obese	− 0.431	− 0.635, − 0.226	0.104	< 0.001	− 0.294	− 0.492, − 0.096	0.101	0.004
Age	− 0.001	− 0.007, 0.006	0.003	0.810	0.007	0.001, 0.013	0.003	0.028
Diabetes mellitus	− 0.189	− 0.341, − 0.037	0.077	0.015	− 0.187	− 0.335, − 0.038	0.076	0.014
	Self-sacrificing vs. self-indulgent				Dislikes food vs. likes food			
(Intercept)	3.279	3.062, 3.496	0.111	< 0.001	4.167	3.855, 4.48	0.159	< 0.001
Gender female (reference male)	0.029	− 0.107, 0.165	0.069	0.673	< 0.001	− 0.194, 0.195	0.099	0.998
Obese	− 0.410	− 0.585, − 0.236	0.089	< 0.001	− 0.322	− 0.571, − 0.073	0.127	0.011
Age	0.005	0, 0.011	0.003	0.050	− 0.006	− 0.013, 0.002	0.004	0.157
Diabetes mellitus	− 0.076	− 0.206, 0.054	0.066	0.252	− 0.081	− 0.267, 0.105	0.095	0.391
	Undereats vs. overeats				Secure vs. insecure			
(Intercept)	3.925	3.641, 4.21	0.145	< 0.001	3.526	3.248, 3.804	0.142	< 0.001
Gender female (reference male)	− 0.008	− 0.186, 0.169	0.090	0.929	0.052	− 0.122, 0.226	0.089	0.556
Obese	− 0.416	− 0.643, − 0.189	0.116	< 0.001	− 0.105	− 0.327, 0.117	0.113	0.353
Age	0.000	− 0.007, 0.007	0.004	0.949	− 0.004	− 0.011, 0.003	0.004	0.275
Diabetes mellitus	− 0.022	− 0.192, 0.148	0.086	0.803	− 0.057	− 0.223, 0.109	0.085	0.500

*B-value*, estimate; *CI*, confidence interval

## Discussion

In the present study, we were able to analyze the influence of different demographic characteristics on stigmatization of obesity using the German FPS short form. Our results show that participants with obesity rated the FPS lower (more positive) than participants without obesity, meaning that they link themselves to more positive characteristics. Gender and age did not have a significant impact on mean FPS ratings. However, when looking at single adjective pairs, female gender was associated with rating individuals with obesity as more attractive and shapelier. Furthermore, those participants that suffered from diabetes mellitus reported lower FPS ratings than their respective counterparts.

A study by Stein et al. defined values < 2.5 as rather positive or neutral and values > 2.5 as rather negative [33]. The mean FPS value of the participants in the present study was rather high (3.5 ± 0.6) indicating an overall negative picture towards obesity. Participants with obesity showed less fat phobia in total and also in most of the single adjective pairs compared with their counterparts without obesity. This is in line with the results from a telephone-based study in Germany, in which a more positive view of obesity was reported when asking individuals with obesity themselves [34]. This study by Sikorski et al. with 3003 participants showed that overweight individuals or those with partners with obesity or overweight reported less negative positions towards obesity. Internal causes such as lack of activity or eating behaviour were frequently named for adults with obesity, whereas external factors such as education or behaviour of the parents were named for children with obesity. The negative stigma that is associated with obesity as a disease is a major barrier for patients and their relatives to accept it as a disease and to seek professional treatment [35, 36]. Puhl et al. and Nickel et al. reported a high degree of stigmatization among healthcare professionals [17, 20, 37]. It has also been reported that obesity is among the most common forms of discrimination besides gender, race and sexual orientation [38]. This shows that even those who should know about obesity as a chronic disease and are often confronted with these patients associate negative characteristics and mainly self-responsibility with obesity. The results from a randomized study with 949 participants by Nickel et al. indicate that interventions or campaigns providing neutral information on obesity to medical professionals and the general population can reduce “fat phobia” and increase the appreciation for obesity as a chronic disease that needs professional treatment [20].

In the present study, there were no differences in gender or age regarding the total fat phobia scale. However, participants that reported to suffer from diabetes mellitus had lower scores (more positive) in the fat phobia scale. In a study by Puhl et al., there were also no differences between males and females regarding types or frequency of stigmatization [39]. However, Nickel et al. reported that female and younger participants regularly rated the burden of obesity and the impact on the daily life higher than their male and older counterparts [20]. The finding that participants that suffer from diabetes mellitus rated people with obesity more positive can be due to the fact many people with obesity present with diabetes mellitus as a comorbidity. Also, people that experiences the impairments and difficulties of having a chronic disease could feel sympathy for others that experience similar diseases.

Looking at the individual adjective pairs, participants with obesity linked the more positive adjective to people with obesity in 12 out of 14 pairs compared with participants with normal weight. Characteristics such as being industrious and having willpower were also more frequently attributed by younger compared with older participants. In other studies that reported on the stigmatization of patients with obesity by practitioners towards, these people were described as lazier and having less willpower than their normal weight counterparts [40]. Characteristics such as “laziness” or “inactivity” are often linked to obesity. Kreuser et al. have reported that indeed children with obesity were less active than their normal weight counterparts, assuming that they did not reach the amount of physical activity to prevent overweight [41]. It was reported people find individuals with obesity less attractive than their normal weight counterparts [42], which was also shown in the present study where participants without obesity attributed the adjective non-attractive more often to participants without obesity. However, these kind of studies may contribute to stereotypes in the general population and other groups [17].

A pooled analysis by the NCD Risk Factor Collaboration involving 19.2 adults discovered a large increase in the prevalence of obesity from 1974 to 2014 [43]. It was recently reported by the National Center of Health Statistics that the prevalence of obesity in US-American adults increased from 30.5 to 42.4% from 1990 through 2017–2018 [1]. This finding foreshadows the influence obesity will have on healthcare systems and emphasizes the importance and need for target-orientated handling of this problem today. Obesity treatment, especially surgical options, can not only help to decrease BMI and improve obesity-related diseases [44–48] but also leads to a higher quality of life [49–53]. However, it has been shown that stigmatization and weight bias can interfere with the ability of patients with obesity to achieve optimal health via treatment options [54]. This is underlined by a study from Sikorski et al. that showed that children with obesity are being stigmatized compared with adults with obesity when using the FPS, even though mostly external factors are considered for paediatric obesity [34]. This highlights the fact that especially children suffer the most of stigmatization even though they are not made responsible themselves. Nickel et al. reported that only patients affected by obesity mention a high need for professional treatment [20].

Because of the impact treatment can have on obesity, it is important to address how healthcare professionals and laymen view and handle patients with obesity. Furthermore, healthcare professionals are supposed to guide people with obesity through the process of losing weight including offering psychological support. This is why it is important that stigmatization does not interfere with professional healthcare [8, 55]. Results from our study indicate that healthcare professionals still stigmatize patients with obesity same the general population would and connects overall negative characteristics with these patients. Furthermore, a different study suggested that some dieticians considered their patients with obesity as “less receptive, less motivated and as having a lower ability to understand and sustain recommendations” [56]. These feelings could be attributed to the lack of proper training. A survey found that Israelian family physicians who commonly saw patients with obesity in clinic felt that they were not adequately trained and had insufficient knowledge regarding nutritional and pharmacologic interventions to help their patients [57]. Another article reports similar results in the USA [58]. Proper training of physicians and nurses may lead to improved stigmatization and ultimately effective treatment of obesity. Even though it has been shown that bariatric surgery is the most effective treatment for long-term weight loss, the general population was shown to classify surgery as non-effective or not recommended and were furthermore unsure of its risks [59–61]. To date, there are only few studies reporting on interventions to reduce weight-related stigmatization [8, 62]. This needs to be a central aim of future studies, especially when considering the discrimination of people with obesity that has been reported in both healthcare and non-healthcare workers in the present study and in other studies.

### Limitations

Most studies using the FPS are based on samples of specific groups in healthcare. However, the mean FPS score was comparable with other studies that assessed other groups such as nutritionists or psychology students. Furthermore, a part of the participants (6.3%) did not answer the FPS completely. People with obesity are stigmatized partly because they are blamed for their condition. The inclusion and comparison of groups where blame is part of the stigmatization may help to differentiate this effect [63]. Even though the fat phobia scale in the short form was developed in 2001 (before the rise of bariatric surgery), it still reflects main characteristics that are connected to people with obesity such as laziness, overeating and non-attractiveness as for example recently published by Robstad et al. [64] and Reddon et al. [65]. These and other studies on the contrary often used self-developed questionnaires which were not validated or were only used in smaller cohorts compared with the fat phobia scale. In order to be able to compare our results with those that have already been published, we therefore decided to use the fat phobia scale for wide comparability.

People who regularly visit outpatient clinics for obesity and medical staff have already received lots of information about this topic and may be biased when answering the FPS. This is particularly true for the patients with obesity that were evaluated in this study at the outpatient clinic. Because of their knowledge about the topic, the patients with obesity in the study may not accurately represent the overall group of people with obesity.

Another limitation is the non-standardized sampling method that was due to the design of this study that aimed to include participants from different professions in the health sector. However, with using linear regression models, a certain standardization towards general demographics was achieved.

## Conclusion

The findings of the present study indicate that fat phobia is generally present in medical professionals as well as the general population. People with obesity are still presumed to have rather negative characteristics associated with them by others. Characteristics such as “laziness” or “inactivity” were linked to patients with obesity in the present study. Especially people that are obese themselves have a more positive picture of individuals with obesity. Gender seems to play a secondary role only regarding certain items of stigmatization. The overall stigmatization of obesity is a major issue as it can greatly impede treatment progress and success for patients with obesity. Negative characteristics that are not true per se are connected more frequently. These findings show the need for both medical professionals and the general population to be informed in detail about the impact, risks and the treatment of obesity in order to effectively decrease the stigmatization and increase the awareness of adequate treatment and prevention. Anti-stigmatization campaigns and other interventions among the general population could help to effectively reduce stigmatization of obesity.
